# Crystal structure of chlorido­{1-(2,3-dimethyl-5-oxido-1-phenyl-1*H*-pyrazol-2-ium-4-yl-κ*O*)-2-[3-methyl-5-oxo-1-phenyl-4,5-di­hydro-1*H*-pyrazol-4-yl­idene-κ*O*]hydrazin-1-ido-κ*N* ^1^}copper(II) from laboratory X-ray powder data

**DOI:** 10.1107/S205698901402756X

**Published:** 2015-01-03

**Authors:** Olga Kovalchukova, Van Nguen, Svetlana Strashnova, Dmitry Kuznetsov, Teimuraz Berikashvili

**Affiliations:** a Peoples’ Friendship University of Russia, 6 Miklukho-Mallaya, 117198 Moscow, Russia; b Moscow State University of Design and Technology, 33 Sadovnicheskaya, 117997 Moscow, Russia; c Georgian Technical University, 77 Kostava, 0175 Tbilisi, Georgia

**Keywords:** crystal structure, azo­pyrazolone, copper complex, powder diffraction

## Abstract

In the title compound, [Cu(C_21_H_19_N_6_O_2_)Cl], the Cu^II^ atom is in a slightly distorted square-planar coordination involving two O atoms from the pyrazolone rings [Cu—O = 2.088 (10) and 1.975 (10) Å], an N atom of the azo group [Cu—N = 2.048 (13) Å] and a chloride anion [Cu—Cl = 2.183 (5) Å]. The organic anions act as tridentate chelating ligands. The mol­ecules stack in columns along the *c* axis.

## Abstract 2

In the title copper(II) complex containing chloride and a derivative of 3-methyl-1-phenyl-4-hydrazopyrazolin-5-one, acting as a tridentate ligand, the Cu^II^ atom is in a slightly distorted square-planar coordination. Mol­ecules stack in columns along the *c* axis.

## Chemical context   

Derivatives of 3-methyl-1-phenyl-4-hydrazopyrazolin-5-one and their metal complexes are well known dyes and possess a wide spectrum of biological activity (Wiley & Wiley, 2008 ▸; Liu *et al.*, 2007 ▸; Hallas & Towns, 1996 ▸). Despite the fact that quite a number of metal complexes are known to exist, the determination of their crystal structures is rather problematic because of the high dispersity of azo-dyes. Only few of them have been structurally characterized (El-Hefnawy *et al.*, 1992 ▸; Casas *et al.*, 2007 ▸; Emeleus *et al.*, 2001 ▸; Zaitseva *et al.*, 1981 ▸; Kovalchukova *et al.*, 2012 ▸; Bansse *et al.*, 1997 ▸; Lalor *et al.*, 1995 ▸). All of them show two similar coordination modes of the organic mol­ecules: bidentate chelating for those with no donating atoms in the aryl­azo fragment or tridentate chelating for ligands with an extra coordinating group.
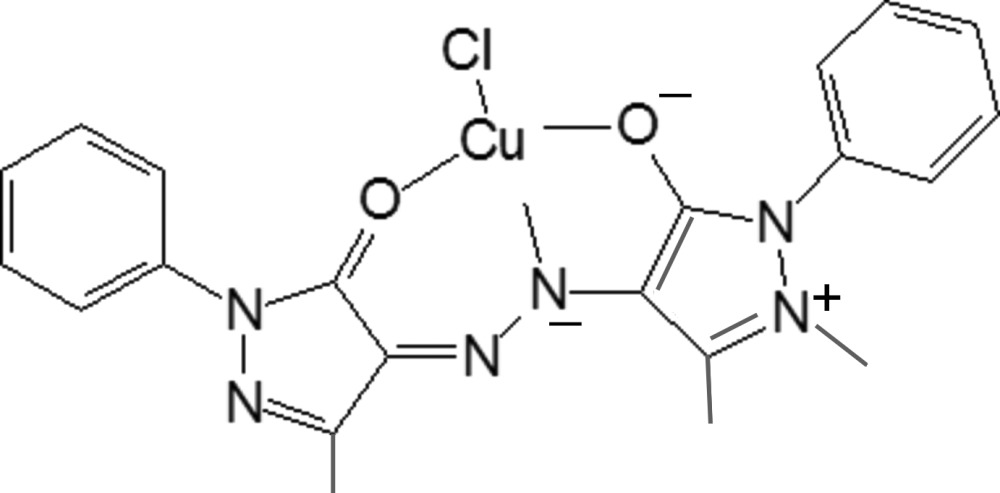


## Structural commentary   

**Figure 1:**
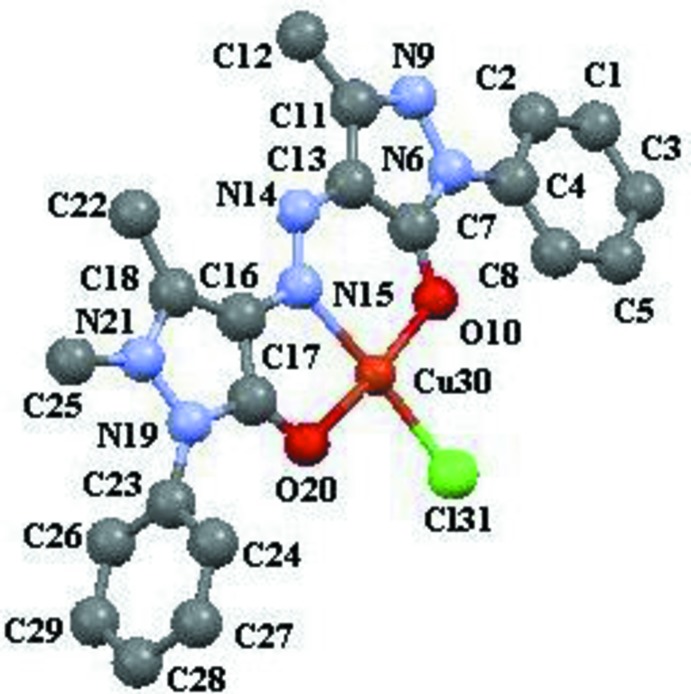
View of the title compound showing the atomic numbering. H atoms are omitted for clarity.

The central Cu^II^ atom is in a square-planar coordination (Fig. 1 ▸) by two O atoms from the pyrazolone rings, an N atom of the azo group, and a chloride anion. The coordination is slightly distorted in view of the two Cu—O bond lengths [2.088 (10) and 1.975 (10) Å], the Cu—Cl [2.183 (5) Å] and the Cu—N bond lengths [2.048 (13) Å]. The sum of the bond angles at the Cu atom [O10—Cu30—N15 = 90.9 (5), O10—Cu30—Cl31 94.0 (4), N15—Cu30—O20 = 83.0 (5), O20—Cu30—Cl31 92.0 (3)°] equals 359.9° which is indicative of the planarity of the Cu^II^ coordination. The organic anions act as tridentate chelating ligands. The N14—N15, N14—C13 and N15—C16 bond lengths [1.306 (17), 1.34 (2) and 1.39 (2) Å, respectively] are very close, thus indicating a strong conjunction of the two pyrazolone fragments which lie in one plane [maximum deviation 0.134 (13) Å for N14]. The benzene rings of the substituents are twisted around this plane by 83 (2) and 9(3)°.

## Supra­molecular features   

**Figure 2:**
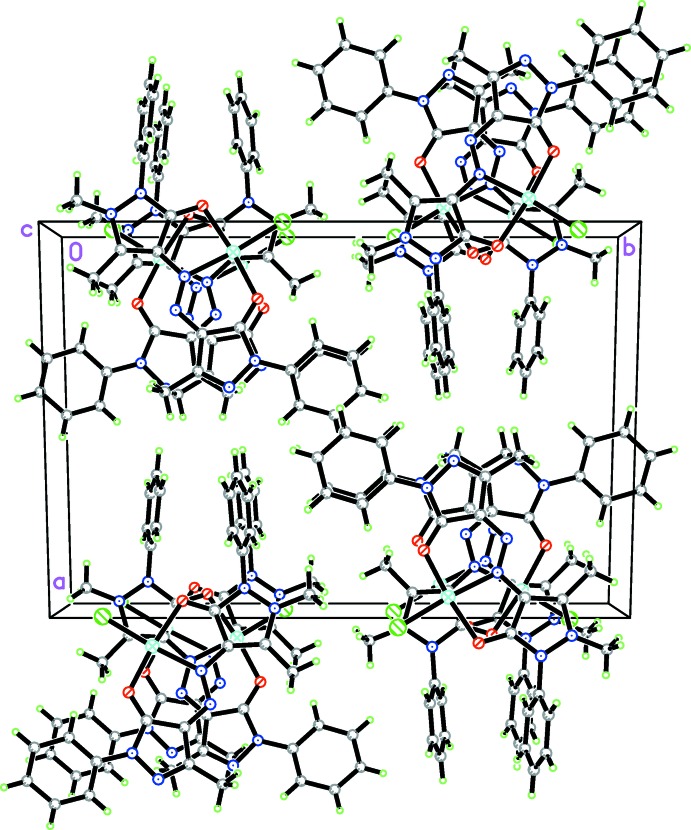
View of the crystal packing along the *b* axis.

In the crystal, the mol­ecules are stacked in columns along the *c* axis in such a way that mol­ecules in neighboring columns at the same level are rotated by approximately 90° (Fig. 2 ▸). No Cu⋯Cu inter­actions between Cu^II^ atoms of neighboring mol­ecules are found.

## Database survey   

The crystal structures of metal complexes with azo­pyrazolone derivatives are described by El–Hefnawy *et al.* (1992 ▸), Casas *et al.* (2007 ▸), Emeleus *et al.* (2001 ▸), Zaitseva *et al.* (1981 ▸), Kovalchukova *et al.* (2012 ▸), Bansse *et al.* (1997 ▸) and Lalor *et al.* (1995 ▸).

## Synthesis and crystallization   

The title compound was prepared by mixing equimolar ethanol solutions of the organic ligand and copper(II) chloride. The reaction mixture was stirred under reflux for three hours. After cooling, fine brown needles of the title complex precipitated. These were then filtered off, washed using a small amount of ethanol and dried over P_2_O_5_.

## Refinement details   

The X-ray powder diffraction data were collected using a Huber G670 Guinier camera (Cu-*K*_α1_ radiation, λ = 1.54059 Å) equipped with an image-plate detector. The monoclinic unit-cell dimensions were determined using three indexing programs: *TREOR90* (Werner *et al.*, 1985 ▸), *ITO* (Visser, 1969 ▸) and *AUTOX* (Zlokazov, 1992 ▸, 1995 ▸). Based on systematic extinctions, the space group was determined to be *P*2_1_/*c*. The unit-cell parameters and space group were further tested using a Pawley (1981 ▸) fit and confirmed by the crystal structure solution.

**Figure 3:**
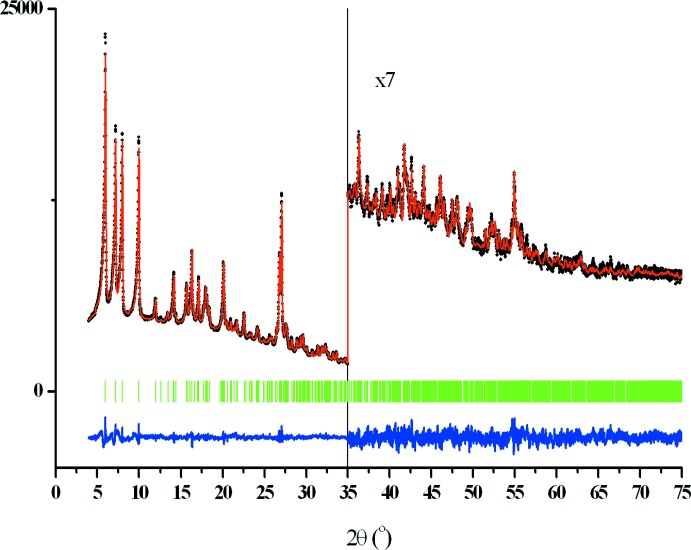
Final Rietveld plot. The experimental diffraction profile is indicated by black dots. The calculated diffraction profile is shown as the top red line, the difference profile is shown as the bottom blue line and the vertical green bars correspond to the positions of the Bragg reflections.

The crystal structure was solved with the use of a simulated annealing technique (Zhukov *et al.*, 2001 ▸). The initial mol­ecular model of the title complex was obtained using density functional theory (DFT) calculations *in vacuo* using the quantum-chemical code *Priroda* (Laikov, 1997 ▸, 2004 ▸, 2005 ▸; Laikov & Ustynyuk, 2005 ▸) employing the generalized-gradient approximation (GGA) and PBE exchange correlation function (Perdew *et al.*, 1996 ▸). In simulated annealing runs (without H atoms), the total number of varied degrees of freedom (DOF) was eight: three translational, three orientational and two torsional ones for the rotation of the two phenyl rings. The solution was fitted with the program *MRIA* (Zlokazov & Chernyshev, 1992 ▸) in a bond-restrained Rietveld refinement using a split-type pseudo-Voigt peak-profile function (Toraya, 1986 ▸) and symmetrized harmonics expansion up to the 4th order (Ahtee *et al.*, 1989 ▸; Järvinen, 1993 ▸) for the texture formalism. Restraints were applied to the intra­molecular bond lengths and contacts (< 2.8 Å) where the strength of the restraints was a function of inter­atomic separation and, for intra­molecular bond lengths, corresponded to an r.m.s. deviation of 0.02 Å. Additional restraints were applied to the planarity of aromatic rings with the attached atoms, with a maximum allowed deviation from the mean plane of 0.03 Å. All non-H atoms were refined isotropically. H atoms were positioned geometrically (C—H = 0.93–0.96 Å) and not refined. The experimental and calculated diffraction profile after the final bond-restrained Rietveld refinements is shown in Fig. 3 ▸. Crystal data, data collection and structure refinement details are summarized in Table 1 ▸.

## Supplementary Material

Crystal structure: contains datablock(s) I, global. DOI: 10.1107/S205698901402756X/vn2088sup1.cif

Rietveld powder data: contains datablock(s) I. DOI: 10.1107/S205698901402756X/vn2088Isup2.rtv

Supporting information file. DOI: 10.1107/S205698901402756X/vn2088Isup3.mol

Supporting information file. DOI: 10.1107/S205698901402756X/vn2088Isup4.mol

CCDC reference: 1040070

Additional supporting information:  crystallographic information; 3D view; checkCIF report

## Figures and Tables

**Table 1:** Experimental details

Crystal data	
Chemical formula	[Cu(C_21_H_19_N_6_O_2_)Cl]
*M*_r_	486.41
Crystal system, space group	Monoclinic, *P*2_1_/*c*
Temperature (K)	298
*a*, *b*, *c* ()	15.1520(18), 22.1306(17), 6.7310(14)
()	101.80(2)
*V* (^3^)	2209.4(6)
*Z*	4
Radiation type	Cu *K*_1_, = 1.54059
(mm^1^)	2.76
Specimen shape, size (mm)	Flat sheet, 15 1

Data collection	
Diffractometer	Guinier camera G670
Specimen mounting	Thin layer in the specimen holder of the camera
Data collection mode	Transmission
Scan method	Continuous
2 values ()	2_min_ = 4.00, 2_max_ = 75.00, 2_step_ = 0.01

Refinement	
*R* factors and goodness of fit	*R*_p_ = 0.019, *R*_wp_ = 0.024, *R*_exp_ = 0.019, *R*_Bragg_ = 0.088, ^2^ = 1.734
No. of data points	7101
No. of parameters	155
No. of restraints	117
H-atom treatment	H-atom parameters not refined

Computer programs: *G670 Imaging Plate Guinier Camera Software* (Huber, 2002 ▸), *MRIA* (Zlokazov Chernyshev, 1992 ▸), (Zhukov *et al.*, 2001 ▸), *PLATON* (Spek, 2009 ▸), and *SHELXL97* (Sheldrick, 2008 ▸).

## supplementary crystallographic information

### Crystal data

**Table:**

[Cu(C_21_H_19_N_6_O_2_)Cl]	*F*(000) = 996
*M**_r_* = 486.41	*D*_x_ = 1.462 Mg m^−^^3^
Monoclinic, *P*2_1_/*c*	Cu *K*α_1_ radiation, λ = 1.54059 Å
Hall symbol: -P 2ybc	µ = 2.76 mm^−^^1^
*a* = 15.1520 (18) Å	*T* = 298 K
*b* = 22.1306 (17) Å	Particle morphology: needle
*c* = 6.7310 (14) Å	brown'
β = 101.80 (2)°	flat sheet, 15 × 1 mm
*V* = 2209.4 (6) Å^3^	Specimen preparation: Prepared at 298 K and 101 kPa
*Z* = 4	

### Data collection

**Table:**

Guinier camera G670 diffractometer	Data collection mode: transmission
Radiation source: line-focus sealed tube	Scan method: continuous
Curved Germanium (111) monochromator	2θ_min_ = 4.00°, 2θ_max_ = 75.00°, 2θ_step_ = 0.01°
Specimen mounting: thin layer in the specimen holder of the camera	

### Refinement

**Table:**

Refinement on *I*_net_	Profile function: split-type pseudo-Voigt (Toraya, 1986)
Least-squares matrix: full with fixed elements per cycle	155 parameters
*R*_p_ = 0.019	117 restraints
*R*_wp_ = 0.024	0 constraints
*R*_exp_ = 0.019	H-atom parameters not refined
*R*_Bragg_ = 0.088	Weighting scheme based on measured s.u.'s
χ^2^ = 1.734	(Δ/σ)_max_ = 0.002
7101 data points	Background function: Chebyshev polynomial up to the 5th order
Excluded region(s): none	Preferred orientation correction: Symmetrized harmonics expansion up to the 4th order (Ahtee *et al.*, 1989; Järvinen, 1993)

### Special details

**Table:**

Geometry. All e.s.d.'s (except the e.s.d. in the dihedral angle between two l.s. planes) are estimated using the full covariance matrix. The cell e.s.d.'s are taken into account individually in the estimation of e.s.d.'s in distances, angles and torsion angles; correlations between e.s.d.'s in cell parameters are only used when they are defined by crystal symmetry. An approximate (isotropic) treatment of cell e.s.d.'s is used for estimating e.s.d.'s involving l.s. planes.

### Fractional atomic coordinates and isotropic or equivalent isotropic displacement parameters (Å^2^)

**Table:**

	*x*	*y*	*z*	*U*_iso_*/*U*_eq_	
C1	0.5220 (10)	0.5094 (8)	0.264 (3)	0.094 (8)*	
H1	0.4633	0.5001	0.2748	0.113*	
C2	0.5471 (9)	0.5696 (8)	0.249 (3)	0.101 (9)*	
H2	0.5048	0.6002	0.2453	0.121*	
C3	0.5843 (10)	0.4630 (7)	0.262 (3)	0.103 (9)*	
H3	0.5669	0.4228	0.2680	0.123*	
C4	0.6367 (10)	0.5838 (8)	0.240 (3)	0.118 (9)*	
C5	0.7003 (10)	0.5375 (7)	0.243 (3)	0.096 (8)*	
H5	0.7599	0.5467	0.2398	0.115*	
N6	0.6604 (8)	0.6455 (6)	0.225 (2)	0.105 (6)*	
C7	0.7447 (11)	0.6707 (8)	0.238 (3)	0.107 (9)*	
C8	0.6730 (9)	0.4775 (8)	0.252 (3)	0.098 (8)*	
H8	0.7145	0.4466	0.2509	0.117*	
N9	0.5931 (9)	0.6898 (6)	0.206 (2)	0.119 (7)*	
O10	0.8175 (6)	0.6407 (5)	0.2551 (17)	0.087 (5)*	
C11	0.6328 (11)	0.7427 (8)	0.206 (2)	0.110 (8)*	
C12	0.5804 (10)	0.8000 (7)	0.191 (3)	0.091 (9)*	
H12A	0.5176	0.7909	0.1805	0.136*	
H12B	0.5882	0.8220	0.0731	0.136*	
H12C	0.6015	0.8240	0.3102	0.136*	
C13	0.7285 (10)	0.7352 (7)	0.224 (2)	0.093 (9)*	
N14	0.7885 (8)	0.7798 (6)	0.226 (2)	0.089 (6)*	
N15	0.8735 (8)	0.7646 (6)	0.2574 (19)	0.101 (7)*	
C16	0.9368 (10)	0.8107 (8)	0.279 (3)	0.106 (9)*	
C17	1.0271 (10)	0.7878 (7)	0.304 (3)	0.095 (9)*	
C18	0.9411 (9)	0.8733 (8)	0.288 (3)	0.101 (9)*	
N19	1.0839 (8)	0.8384 (6)	0.327 (2)	0.093 (7)*	
O20	1.0518 (6)	0.7337 (5)	0.3047 (16)	0.094 (6)*	
N21	1.0321 (8)	0.8895 (6)	0.319 (2)	0.104 (7)*	
C22	0.8697 (10)	0.9203 (8)	0.268 (3)	0.099 (8)*	
H22A	0.8971	0.9596	0.2829	0.149*	
H22B	0.8354	0.9145	0.3720	0.149*	
H22C	0.8306	0.9171	0.1373	0.149*	
C23	1.1789 (10)	0.8343 (7)	0.394 (3)	0.106 (9)*	
C24	1.2315 (10)	0.8204 (7)	0.251 (3)	0.097 (9)*	
H24	1.2047	0.8152	0.1148	0.116*	
C25	1.0666 (10)	0.9483 (7)	0.346 (3)	0.095 (9)*	
H25A	1.1311	0.9470	0.3641	0.143*	
H25B	1.0502	0.9661	0.4631	0.143*	
H25C	1.0421	0.9720	0.2280	0.143*	
C26	1.2192 (11)	0.8450 (7)	0.598 (3)	0.111 (9)*	
H26	1.1841	0.8549	0.6911	0.133*	
C27	1.3248 (10)	0.8145 (7)	0.316 (3)	0.098 (9)*	
H27	1.3601	0.8038	0.2238	0.118*	
C28	1.3126 (10)	0.8405 (7)	0.658 (3)	0.108 (9)*	
H28	1.3400	0.8482	0.7924	0.130*	
C29	1.3654 (11)	0.8246 (7)	0.519 (3)	0.110 (9)*	
H29	1.4275	0.8207	0.5619	0.132*	
Cu30	0.93520 (16)	0.68180 (13)	0.2845 (5)	0.0760 (14)*	
Cl31	1.0116 (3)	0.5976 (2)	0.3067 (8)	0.084 (2)*	

### Geometric parameters (Å, º)

**Table:**

C1—C2	1.40 (2)	C16—C17	1.44 (2)
C1—C3	1.40 (2)	C17—O20	1.255 (19)
C1—H1	0.93	C17—N19	1.40 (2)
C2—C4	1.41 (2)	C18—N21	1.398 (18)
C2—H2	0.93	C18—C22	1.49 (2)
C3—C8	1.40 (2)	N19—N21	1.372 (18)
C3—H3	0.93	N19—C23	1.420 (19)
C4—C5	1.40 (2)	O20—Cu30	2.088 (10)
C4—N6	1.42 (2)	N21—C25	1.40 (2)
C5—C8	1.40 (2)	C22—H22A	0.96
C5—H5	0.93	C22—H22B	0.96
N6—C7	1.38 (2)	C22—H22C	0.96
N6—N9	1.401 (19)	C23—C24	1.40 (3)
C7—O10	1.27 (2)	C23—C26	1.40 (3)
C7—C13	1.45 (2)	C24—C27	1.40 (2)
C8—H8	0.93	C24—H24	0.93
N9—C11	1.32 (2)	C25—H25A	0.96
O10—Cu30	1.975 (10)	C25—H25B	0.96
C11—C13	1.44 (2)	C25—H25C	0.96
C11—C12	1.49 (2)	C26—C28	1.39 (2)
C12—H12A	0.96	C26—H26	0.93
C12—H12B	0.96	C27—C29	1.40 (3)
C12—H12C	0.96	C27—H27	0.93
C13—N14	1.34 (2)	C28—C29	1.39 (3)
N14—N15	1.306 (17)	C28—H28	0.93
N15—C16	1.39 (2)	C29—H29	0.93
N15—Cu30	2.048 (13)	Cu30—Cl31	2.183 (5)
C16—C18	1.39 (2)		
			
C2—C1—C3	120.5 (15)	N19—C17—C16	106.3 (13)
C2—C1—H1	119.7	C16—C18—N21	107.4 (13)
C3—C1—H1	119.8	C16—C18—C22	131.9 (14)
C1—C2—C4	119.7 (15)	N21—C18—C22	120.7 (14)
C1—C2—H2	120.1	N21—N19—C17	108.7 (12)
C4—C2—H2	120.1	N21—N19—C23	126.9 (12)
C1—C3—C8	119.4 (15)	C17—N19—C23	122.9 (13)
C1—C3—H3	120.3	C17—O20—Cu30	106.1 (9)
C8—C3—H3	120.3	N19—N21—C18	109.5 (12)
C5—C4—C2	120.1 (16)	N19—N21—C25	124.4 (12)
C5—C4—N6	121.3 (14)	C18—N21—C25	126.1 (13)
C2—C4—N6	118.6 (15)	C18—C22—H22A	109.5
C8—C5—C4	119.1 (15)	C18—C22—H22B	109.5
C8—C5—H5	120.5	H22A—C22—H22B	109.4
C4—C5—H5	120.4	C18—C22—H22C	109.5
C7—N6—N9	111.7 (13)	H22A—C22—H22C	109.5
C7—N6—C4	128.9 (13)	H22B—C22—H22C	109.4
N9—N6—C4	119.3 (12)	C24—C23—C26	120.8 (15)
O10—C7—N6	124.7 (15)	C24—C23—N19	118.6 (15)
O10—C7—C13	130.7 (15)	C26—C23—N19	120.6 (16)
N6—C7—C13	104.6 (13)	C27—C24—C23	119.0 (17)
C5—C8—C3	121.1 (15)	C27—C24—H24	120.5
C5—C8—H8	119.4	C23—C24—H24	120.5
C3—C8—H8	119.5	N21—C25—H25A	109.5
C11—N9—N6	107.4 (13)	N21—C25—H25B	109.5
C7—O10—Cu30	121.1 (10)	H25A—C25—H25B	109.5
N9—C11—C13	110.5 (15)	N21—C25—H25C	109.5
N9—C11—C12	121.3 (14)	H25A—C25—H25C	109.4
C13—C11—C12	128.2 (15)	H25B—C25—H25C	109.5
C11—C12—H12A	109.5	C28—C26—C23	119.1 (17)
C11—C12—H12B	109.5	C28—C26—H26	120.4
H12A—C12—H12B	109.5	C23—C26—H26	120.5
C11—C12—H12C	109.5	C24—C27—C29	120.5 (18)
H12A—C12—H12C	109.5	C24—C27—H27	119.8
H12B—C12—H12C	109.5	C29—C27—H27	119.8
N14—C13—C11	125.8 (15)	C29—C28—C26	120.7 (16)
N14—C13—C7	128.3 (14)	C29—C28—H28	119.7
C11—C13—C7	105.9 (14)	C26—C28—H28	119.7
N15—N14—C13	117.2 (13)	C28—C29—C27	119.9 (14)
N14—N15—C16	117.7 (13)	C28—C29—H29	120.1
N14—N15—Cu30	131.5 (10)	C27—C29—H29	120.1
C16—N15—Cu30	110.8 (10)	O10—Cu30—N15	90.9 (5)
N15—C16—C18	139.8 (15)	O10—Cu30—O20	173.8 (4)
N15—C16—C17	112.0 (14)	N15—Cu30—O20	83.0 (5)
C18—C16—C17	108.2 (14)	O10—Cu30—Cl31	94.0 (4)
O20—C17—N19	125.9 (13)	N15—Cu30—Cl31	174.8 (4)
O20—C17—C16	127.9 (14)	O20—Cu30—Cl31	92.0 (3)
